# 52 Open-Label, Expanded-Access Study of a Bioengineered Allogeneic Cellularized Construct in Adults With Deep Partial-Thickness Burns

**DOI:** 10.1093/jbcr/irad045.026

**Published:** 2023-05-15

**Authors:** Angela Gibson, Victor Joe, Joshua Carson, Jeff Litt, Jeffrey Carter, James Holmes

**Affiliations:** University of Wisconsin School of Medicine and Public Health, Madison, Wisconsin; UCI Health, Orange, California; Loyola University Medical Center, Maywood, Illinois; BRCA/Chippenham Hospital, Richmond, Virginia; University Medical Center New Orleans & LSUHSC, River Ridge, Louisiana; Wake Forest School of Medicine, Winston-Salem, North Carolina; University of Wisconsin School of Medicine and Public Health, Madison, Wisconsin; UCI Health, Orange, California; Loyola University Medical Center, Maywood, Illinois; BRCA/Chippenham Hospital, Richmond, Virginia; University Medical Center New Orleans & LSUHSC, River Ridge, Louisiana; Wake Forest School of Medicine, Winston-Salem, North Carolina; University of Wisconsin School of Medicine and Public Health, Madison, Wisconsin; UCI Health, Orange, California; Loyola University Medical Center, Maywood, Illinois; BRCA/Chippenham Hospital, Richmond, Virginia; University Medical Center New Orleans & LSUHSC, River Ridge, Louisiana; Wake Forest School of Medicine, Winston-Salem, North Carolina; University of Wisconsin School of Medicine and Public Health, Madison, Wisconsin; UCI Health, Orange, California; Loyola University Medical Center, Maywood, Illinois; BRCA/Chippenham Hospital, Richmond, Virginia; University Medical Center New Orleans & LSUHSC, River Ridge, Louisiana; Wake Forest School of Medicine, Winston-Salem, North Carolina; University of Wisconsin School of Medicine and Public Health, Madison, Wisconsin; UCI Health, Orange, California; Loyola University Medical Center, Maywood, Illinois; BRCA/Chippenham Hospital, Richmond, Virginia; University Medical Center New Orleans & LSUHSC, River Ridge, Louisiana; Wake Forest School of Medicine, Winston-Salem, North Carolina; University of Wisconsin School of Medicine and Public Health, Madison, Wisconsin; UCI Health, Orange, California; Loyola University Medical Center, Maywood, Illinois; BRCA/Chippenham Hospital, Richmond, Virginia; University Medical Center New Orleans & LSUHSC, River Ridge, Louisiana; Wake Forest School of Medicine, Winston-Salem, North Carolina

## Abstract

**Introduction:**

An FDA-approved bioengineered allogeneic cellularized construct (BACC) provides burn surgeons with an alternative treatment for deep partial-thickness (DPT) burns and may reduce the need for autografting. A phase 3b, open-label, single-arm, multicenter, expanded-access study (NCT04123548) evaluated safety and clinical outcomes of BACC treatment in adults with DPT thermal burns containing intact dermal elements.

**Methods:**

Patients aged ≥18 years with 3% to < 50% total body surface area of partial-thickness ± full-thickness thermal burns were enrolled. A single application of up to 1:1 meshed BACC was applied to ≤3 burn areas on the torso or extremities (≤2000 cm^2^ area of BACC). Patients were followed for 24 weeks. The primary endpoint was count and percentage of patients with treatment-emergent adverse events (TEAEs). Additional endpoints were patients and treatment sites with confirmed wound closure (complete re-epithelialization without drainage for 2 consecutive visits ≥2 weeks apart) of the BACC treatment site without autografting by Week 12; durable wound closure by Week 24; time to wound closure without autografting (determined by Kaplan-Meier estimate); scar evaluation using the Patient and Observer Scar Assessment Scale (POSAS); and patients with wound infection-related events. The sample was not designed or sized to determine efficacy.

**Results:**

Overall, 52 patients were enrolled. Pruritus was the most frequently reported TEAE (n=22 [42.3%]). There were 2 (3.8%) BACC-related TEAEs; both were pruritus and mild in severity. There were 20 serious AEs in 10 (19.2%) patients; none were related to BACC. No patients discontinued the study due to TEAEs. There were 4 (7.7%) deaths (aspiration, myocardial infarction, self-injury, and Gram-negative rod sepsis); none were related to BACC. Wound infection-related events occurred in 5 (9.6%) patients. Overall, 46 (88.5%) patients and 87 (90.6%) BACC-treated wounds did not require autografting. Half of patients achieved wound closure by Week 5. At Weeks 12 and 24, observer and patient total POSAS scores (mean ± SD) were 18.7 ± 8.08 and 18.8 ± 9.84, and 24.5 ± 11.0 and 20.2 ± 12.5, respectively.

Wound closure of the BACC treatment site without autografting

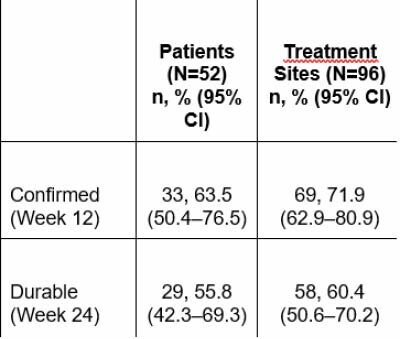

**Conclusions:**

There were no unexpected wound or BACC-related events. The safety of BACC supported its prescribing information. Patients treated with BACC achieved durable wound closure without autografting and thus showed expected clinical benefit in this population. The use of BACC in DPT burns in this study was donor-site sparing.

**Applicability of Research to Practice:**

Treatment with BACC may offer a promising alternative to autografting for DPT burns.

